# Supplementary material for: From sequence to enzyme mechanism using multi-label machine learning
Source: BMC Bioinformatics. 2014 May 19;15:150. doi: 10.1186/1471-2105-15-150 (PMC4229970; doi:10.1186/1471-2105-15-150)
Supplement: Additional file 2 — Java code of ml2db. Additional file ml2db_code.tar.gz contains the Java source code to run the multi-label machine learning experiments and save the results to database. The code’s Javadoc is included. [file 1471-2105-15-150-S2.zip › additional file 2/ml2db/ml2db/doc/overview-frame.html]

Overview List


All Classes

## Packages

- main.ml2db
- main.ml2db.al
- main.ml2db.al.database
- main.ml2db.al.learning
- main.ml2db.al.utility
- main.ml2db.al.utility.confidence
- main.ml2db.al.utility.globaldistance
- main.ml2db.al.utility.hybrid
- main.ml2db.al.utility.neighbours
- main.ml2db.al.utility.pickers
- main.ml2db.al.utility.pickers.byfrequency
- main.ml2db.graphviz
- main.ml2db.mulan.attributesfiltered
- main.ml2db.mulan.classifier.lazy
- main.ml2db.mulan.learn
- main.ml2db.mulan.learn.crosseval
- main.ml2db.mulan.learn.evaluation
- main.ml2db.mulan.learn.traintest
- main.ml2db.mulan.measuremetrics
- main.ml2db.mulan.mulanarff
- main.ml2db.mulan.mulanarff.database
- main.ml2db.mulan.predict
- main.ml2db.weka
- test.ml2db
- test.ml2db.al
- test.ml2db.al.utility.confidence
- test.ml2db.al.utility.globaldistance
- test.ml2db.al.utility.hybrid
- test.ml2db.al.utility.neighbourhood
- test.ml2db.al.utility.pickers
- test.ml2db.alltests
- test.ml2db.dataharness
- test.ml2db.graphviz
- test.ml2db.mulan.attributesfilter
- test.ml2db.mulan.classifier.lazy
- test.ml2db.mulan.learn
- test.ml2db.mulan.learn.database
- test.ml2db.mulan.learn.traintest
- test.ml2db.mulan.measuremetrics
- test.ml2db.mulan.mulanarff
- test.ml2db.mulan.mulanarff.database
- test.ml2db.mulan.predict
- test.ml2db.weka
